# Exploratory analysis of biofilm formation and virulence gene expression in *Acinetobacter baumannii*–*Candida albicans* co-cultured isolates from urinary tract infections

**DOI:** 10.1038/s41598-026-59824-w

**Published:** 2026-07-10

**Authors:** Bassant Hossam, Refaat M. Gabre, Dina Amr, Hazem H. Saleh

**Affiliations:** 1 Department of Biotechnology, Faculty of Science, Cairo University, Giza, Egypt; 2 Urology and Nephrology Centre, Mansoura University, Mansoura, Egypt

**Keywords:** *Acinetobacter baumannii*, *Candida albicans*, Co-culture, Biofilm formation, Virulence genes, Antimicrobial resistance, Diseases, Microbiology

## Abstract

Polymicrobial infections with *Acinetobacter baumannii (A. baumannii)* and *Candida albicans* (*C. albicans*) are increasingly frequent in urinary tract infections (UTIs). However, experimental data describing their interactions in clinical isolates under co-culture conditions remain limited. In this study, three clinical isolates of both *A. baumannii* and *C. albicans* were co-isolated from urine samples with UTIs, then mono-cultured and co-cultured at 24, 48, and 72 h for biofilm quantification by crystal violet assay. The expression levels of bacterial (*ompA, bap, abaI*) and fungal (*ALS3, HWP1, ERG11*) virulence genes were evaluated by RT-qPCR at 24 and 48 h. Co-culture conditions resulted in increased biofilm biomass compared to monoculture for the tested isolates. In *A. baumannii,* the virulence genes of *bap, abaI, and ompA* showed statistically significant increase in expression in co-culture compared to mono-culture after 24 h. In *C. albicans*, *HWP1* is the only virulence gene that shows a statistically significant increase in expression in co-culture compared to monoculture after 48 h. Gene expression patterns varied in patient isolates, suggesting strain heterogeneity. This is an exploratory study that provides evidence of changes in biofilm formation and virulence gene expression in co-culture conditions among clinical isolates of *A. baumannii* and *C. albicans*. These findings suggest potential for microbial interactions under polymicrobial conditions, which might affect the diagnosis and treatment of patients with UTIs. Future studies with a larger number of isolates and functional assays are required to clarify the mechanistic regulation and biological relevance of these observations in UTIs.

**Supplementary Information:**

The online version contains supplementary material available at 10.1038/s41598-026-59824-w.

## Introduction

Microbes in the human body are widely recognised to form complex polymicrobial communities. These communities also apply to most chronic infections, including abscesses, chronic wounds, periodontitis, actinomycosis, pneumonia, and cystic fibrosis, where microbe–microbe interactions increase disease severity^1^. Microbes utilise a wide range of strategies in complex biofilms to shift the balance from colonisation to infection of the host via mechanisms such as cooperation, immune modulation, and cross-feeding^2^. Urinary tract infections (UTIs) are one of the most prevalent infections in humans, affecting about 150 million people annually^3^. Around 60% of women experience UTIs at least once, and 20–30% face recurrence within six months^4^. Antibiotic resistance increases significantly in polymicrobial UTIs, leading to treatment challenges^3^. Multiple studies have demonstrated the presence of identical microorganisms in polymicrobial bloodstream and urinary infections^5–7^. Moreover, polymicrobial UTIs are more common among compromised patients. These polymicrobial UTIs are commonly due to uropathogenic bacteria, such as multi-drug resistant (MDR) *Acinetobacter baumannii, Klebsiella, Enterococcus, Escherichia coli,* and *Staphylococcus*^8^*.* Urogenital infections are also caused by parasites such as *Schistosoma haematobium* and fungi such as *Candida* species^9^. *C. albicans* is the most significant opportunistic fungus leading to nosocomial UTIs^10^. Among Gram-negative bacteria, *Acinetobacter* spp. are identified in 2–61% of nosocomial UTI cases^11^, often associated with urinary catheters and nephrostomy tubes in intensive care unit (ICU) patients, where 17.1% of urinary isolates are *A. baumannii*^12,13^. The clinical significance and epidemiology of polymicrobial infections in urine cultures remain challenging, especially in catheterised patients, with higher mortality rates in polymicrobial than monomicrobial UTI cases^14^. In the ICU, *A. baumannii* often coexists with *C. albicans* in the hospital environment^15,16^. In *A. baumannii*, biofilm formation and pathogenicity are associated with several virulence-related genes and their corresponding protein products. The *ompA* gene encodes outer membrane protein A (OmpA), which contributes to adhesion to host cells and abiotic surfaces. The *bap* gene encodes biofilm-associated protein (Bap), which is involved in biofilm maturation and structural stability. In addition, *abaI* encodes a quorum-sensing synthase (AbaI), which is involved in the synthesis of acyl-homoserine lactone signalling molecules associated with cell–cell communication and regulation of biofilm architecture^17–19^. The *ALS3* gene encodes Als3, a hypha-associated adhesin involved in epithelial adhesion and invasion, whereas the *HWP1* gene encodes Hwp1, a hyphal wall protein important for stable attachment and biofilm development. In addition, the *ERG11* gene encodes lanosterol 14-α-demethylase (Erg11), an enzyme involved in ergosterol biosynthesis and a major target of azole antifungal agents. Alterations in *ERG11* expression have been associated with reduced azole susceptibility and biofilm resilience^20–23^. Therefore, the relative expression levels of these genes were evaluated in the present study using RT-qPCR to investigate virulence and biofilm-associated responses during the interaction between *A. baumannii* and *C. albicans* under co-culture conditions.

## Materials and methods

### In vitro analysis of co-culture between *A. baumannii *and *C. Albicans*

#### Sample collection and isolation

During December 2024 and March 2025, three pairs of clinical isolates of *A. baumannii* and *C. albicans* were co-isolated from urine samples obtained from three independent UTI patients at the Urology and Nephrology Centre (UNC), Mansoura University Hospitals, Dakahlia governorate, Egypt. In the present study, each *A. baumannii* isolate and its corresponding *C. albicans* isolate were obtained from the same UTI patient sample. The research proposal was approved by the Medical Research Ethics Committee Institutional Review Board at the Mansoura Faculty of Medicine, Mansoura University (Code Number: MS.25.11.3375). All methods were performed in accordance with the relevant guidelines and regulations. Urine specimens were cultured on CLED agar and blood agar (Oxoid Ltd., Basingstoke, UK) to recover Gram-negative bacteria and on Sabouraud dextrose agar (SDA) (Oxoid Ltd., Basingstoke, UK) to isolate *C. albicans*. For confirmatory identification and antimicrobial susceptibility testing, isolates were processed using the VITEK 2 compact system (bioMérieux, France) with the GN ID and AST card (GN73) for the Gram-negative bacteria and the YST ID and AST card (Y508) for yeast, following the manufacturer’s instructions^24^.

#### Culture and experimental conditions

Before each experiment, the *C. albicans* isolates were cultured at 37 °C for 48 h on Sabouraud dextrose agar, while *A. baumannii* was sub-cultured at 37 °C for 24 h on tryptic soy agar (TSA) (Oxoid Ltd., Basingstoke, UK). Each strain was then inoculated into 5 ml of tryptic soy broth (TSB) (Oxoid Ltd., Basingstoke, UK) and incubated aerobically at 37 °C for 24 h. Microbial suspensions were adjusted for turbidity to a McFarland 1.0 standard^25^.

#### Biofilm cultivation

Biofilms were grown in 96-well polystyrene flat-bottom microtiter plates (Nest Biotechnology Co., Ltd., Wuxi, China). For each isolate, three microplates were prepared, each incubated for 24, 48, or 72 h at 37 °C. TSB was selected as the culture medium in this study due to its non-selective composition. Moreover, several studies examining dual-species biofilms, including *S. aureus* and *C. albicans*, have successfully employed TSB as a standard medium to support reproducible biofilm development^26,27^. Each condition was tested in technical quadruples (*n* = 4 wells) per isolate: (i) control medium (150 µL TSB), (ii) single-species suspensions (150 µL of either *C. albicans* or *A. baumannii*), or (iii) mixed-species suspensions (75 µL of *C. albicans* plus 75 µL of *A. baumannii*, total 150 µL). Two additional wells were prepared for RNA extraction and RT-qPCR. Microtiter plates were incubated under static conditions without medium replacement and were covered to minimise evaporation. After incubation, biofilm biomass was quantified using the crystal violet (CV) assay^28^.

#### Crystal violet assay

The supernatant broth was aspirated, and biofilms were washed once with 150 µL of 1 × phosphate-buffered saline (PBS, pH 7.2). Then, they were air-dried and stained with 150 µL of 0.1% (w/v) CV (BDH Chemicals Ltd., Poole, UK) for 15 min. Excess CV was removed, and the remaining CV was rinsed twice with 150 µL of 1 × PBS. Fixation was done with 150 µL of 95% absolute ethanol (Merck KGaA, Darmstadt, Germany). Absorbance was read at 620 nm using an ELISA reader (Tecan Group Ltd., Männedorf, Switzerland).

#### Primer design, RNA isolation, and qPCR

*Primer design*: Gene targets and primer sequences used in this study are listed in Table 1. Housekeeping genes used for normalisation were *16S* rRNA for *A. baumannii* and *ACT1* for *C. albicans*. NCBI Primer-BLAST was used to evaluate the specificity of housekeeping gene primer sequences in silico^29^. The *16S* rRNA primer was tested against the *A. baumannii* ATCC 19,606 reference sequence (NR_119358.1), while the *ACT1* primer was tested against the *C. albicans* reference strain CBS 562 NT (AJ389057.1). Primer pairs showed specificity for the intended targets in both species. In addition, no other targets were detected when databases were restricted to the respective species. Amplification efficiency was also verified for each primer pair by melting curve analysis, as shown in the supplementary material. *RNA isolation*: Biofilms of *C. albicans*, *A. baumannii*, and their co-culture were scraped from the wells using a sterile pipette tip, and total RNA, including small RNAs, was extracted using the miRNeasy Mini Kit (Cat. No. 217004; Qiagen GmbH, Hilden, Germany), following the manufacturer’s protocol optimised for fungal and bacterial samples^30^. RNA concentration and purity were assessed using a NanoDrop spectrophotometer (NanoPhotometer N60 Touch, IMPLEN GmbH, Munich, Germany). Samples with a 260/280 ratio of 1.9–2.1 were used for further analyses. *cDNA synthesis*: 500 ng of total RNA from each sample was reverse-transcribed using RScript Reverse Transcriptase (Bio-Helix, Taiwan) according to the manufacturer’s instructions. *RT-qPCR*: Quantitative real-time PCR was performed in technical duplicates using PanGreen Universal SYBR Green Master Mix (Bio-Helix, Taiwan) on a CFX96™ Real-Time PCR Detection System (Bio-Rad Laboratories, Hercules, CA, USA). Each qPCR reaction was performed in a final volume of 20 µL containing 10 µL SYBR Green Master Mix, 0.5 µM of each primer, and cDNA template. The thermal profile was adjusted at the following temperatures: (i) initial activation (5 min at 95 °C), (ii) denaturation (45 cycles of 95 °C for 10 s), (iii) annealing (15 s). RT-qPCR analysis was performed using co-isolates of *A. baumannii* and *C. albicans*. For each sample, reactions were run in technical duplicate to ensure measurement consistency. Mean Ct values of technical replicates were calculated and used for downstream relative expression analysis. Statistical comparisons were performed using biological replicates (*n* = 3 per condition), which were treated as three independent data points. No-template and no-RT controls were included.

**Table 1:** Primer sequences used for qPCR analysis of virulence and housekeeping genes in *A. baumannii* and *C. albicans.*

	Target gene	Primer sequence (5′ → 3′)	Product size (bp)	Tm (°C )	References
*A. baumannii*	*bap*	F: AATGCACGGTACTTGATCC R: TATTGC CTCAGGGT CAGTT	205	51	^31^
	*ompA*	F: ATGAAAAAGACAGCTAT CGC GATTGCA R: CACCAAAAGCAC CAGCGCCCAGTTG	136	55	
	*abaI*	F: AATGCCTAT TCCCT GCT CAC R: ATTGCTT CTTGCAGAATTGC	132	48	
	*16S* rRNA	F: ACTCCTACGGGAGGCAGCAGT R: TAT TACCG CGGCTGCT GGC	198	58	
*C. albicans*	*HWP1*	F: CAGCCACTGAAACACCAACT R: CAGAAGTAACAACAACACCAG	153	50	^32^
	*ERG11*	F: ATTGTTGAAACTGTCATTG R: CCCTTAATATATACTGATCTG	185	41	
	*ALS3*	F: CTAATGCTGCTACGTATAATT R: CCTGAAATTGACATGTAGCA	201	43	
	*ACT1*	F: AGCCCAATCCAAAAGGAGTATT R: GCTTCGGTCAAACAAACTGG	153	50	

#### Statistical analysis

Biofilm biomass data from the crystal violet assay were analysed using the microtiter plate assay method^33^. The cut-off OD (ODc) was calculated as ODc = mean OD of the negative control + (3 × SD), yielding 0.0786; isolates were categorised as non-weak, moderate, or strong biofilm formers accordingly. For each isolate and condition, measurements were performed in quadruplicate technical wells, and the results were averaged before analysis. Statistical analyses were performed using GraphPad Prism 10.5.0 (GraphPad Software, San Diego, CA, USA). Data normality was assessed using the Shapiro–Wilk test. Differences in biofilm biomass were evaluated using one-way ANOVA with Šídák’s post-hoc test, with results interpreted in an exploratory context. Šídák’s multiple comparison test was used for pairwise comparisons between groups, while Dunnett’s test was applied for comparisons against a single reference condition (24 h) in time-course analyses. For RT-qPCR, reactions were run in technical duplicates and averaged before analysis. Statistical comparisons were performed on ΔCt values using paired t-tests, while relative expression levels were calculated using the 2^ − ΔΔCt method for visualisation purposes only^34^. Results are presented as mean ± SD of biological replicates, with *p* < 0.05 considered statistically significant at a 95% confidence level.

## Results

### VITEK-2 antimicrobial susceptibility testing

Antimicrobial susceptibility testing was performed using the VITEK-2 system (bioMérieux, France) for the isolates of *C. albicans* and *A. baumannii*. *C. albicans* isolates were susceptible to all antifungal agents tested. MIC values were within the susceptible range (Table 2). On the other hand, isolates of *A. baumannii* displayed extensive drug resistance (XDR), with resistance detected against all antimicrobial classes tested, and interpretation was based on Clinical and Laboratory Standards Institute (CLSI) 31st edition, 2021 guidelines^35^ (Table 3).

**Table 2:** Antifungal susceptibility profile of *C. albicans* by the VITEK-2 system.

Antifungal agent	MIC (µg/mL)	Interpretation
Voriconazole	≤ 0.12	S
Fluconazole	≤ 0.5	S
Micafungin	≤ 0.06	S
Caspofungin	≤ 0.12	S
Amphotericin B	1	S
Flucytosine	≤ 1	S

*Antifungal classes*: Voriconazole and fluconazole (triazoles); micafungin and caspofungin (echinocandins); amphotericin B (polyene); flucytosine (pyrimidine analogue). *Interpretation*: S: Susceptible; MIC: Minimum Inhibitory Concentration.

**Table 3:** Antimicrobial susceptibility profile of *A. baumannii* by the VITEK-2 system.

Antimicrobial agent	MIC (µg/mL)	Interpretation
Ampicillin/Sulbactam	≥ 32	R
Piperacillin/Tazobactam	≥ 128	R
Cefazolin^†^	≥ 16	R
Ceftazidime	≥ 32	R
Ceftriaxone^†^	≥ 64	R
Cefepime	≥ 64	R
Meropenem	≥ 16	R
Gentamicin	≥ 16	R
Tobramycin	≥ 16	R
Ciprofloxacin	≥ 4	R
Levofloxacin	≥ 4	R

*Antibiotic classes*: Ampicillin/sulbactam and piperacillin/tazobactam (β-lactam/β-lactamase inhibitors); cefazolin, ceftazidime, ceftriaxone, cefepime (cephalosporins); meropenem (carbapenem); gentamicin and tobramycin (aminoglycosides); ciprofloxacin and levofloxacin (fluoroquinolones). *Interpretation*: R: Resistant; MIC: Minimum inhibitory concentration.

### Biofilm quantification assay

#### The difference in biofilm biomass between co-culture and mono-culture

**Fig. 1:**
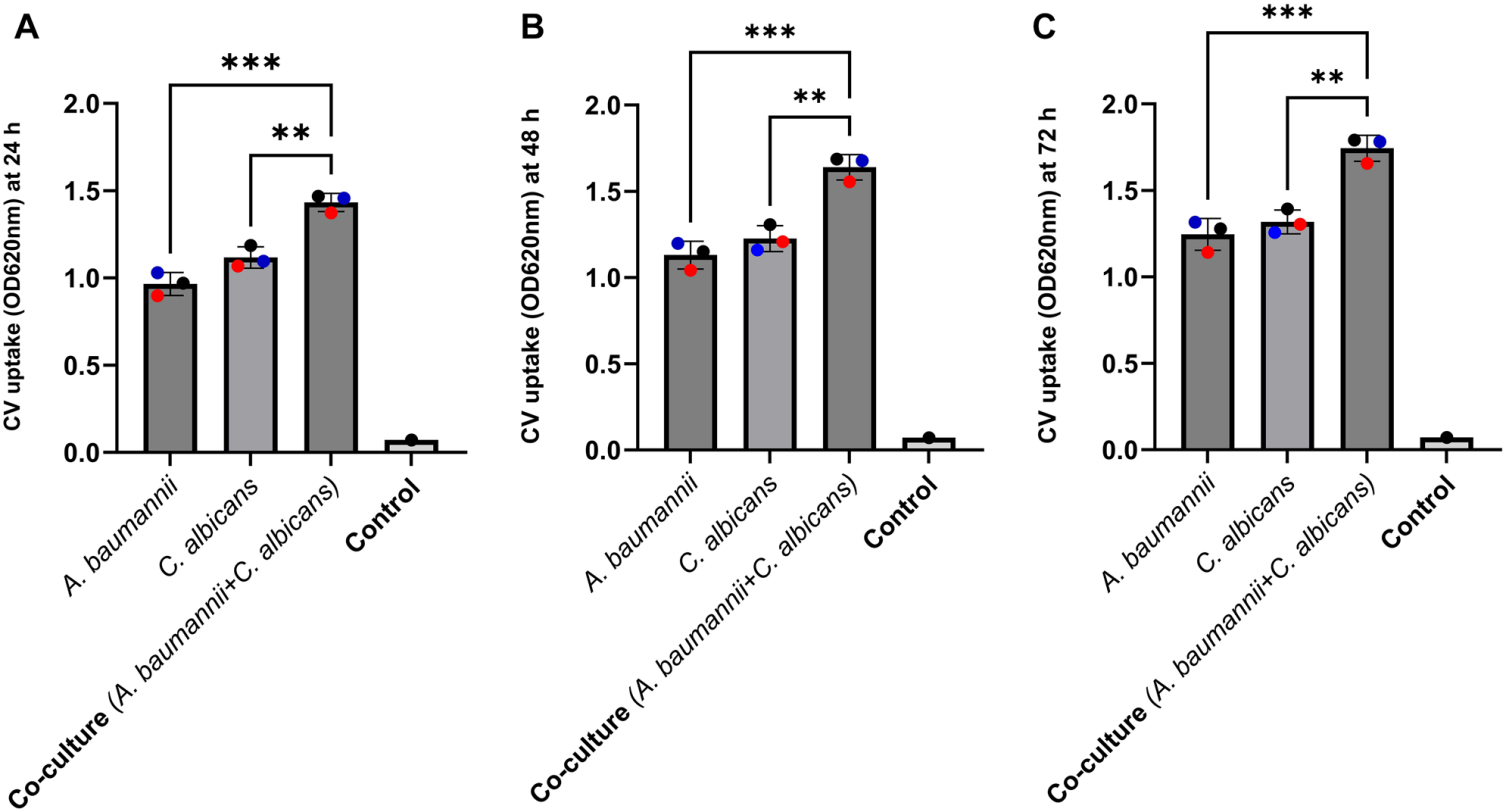
Biofilm biomass was quantified using the CV assay at OD620 nm at (**A**) 24 h, (**B**) 48 h, and (**C**) 72 h of incubation. Data represent the mean ± SD of three independent isolates (blue, red, black dots) per group. Statistical analysis was performed using One-way ANOVA followed by Šídák’s multiple comparison test. Statistical significance is indicated as follows: (*p-value < 0.05, **p-value < 0.01, ***p-value < 0.001).

Crystal violet staining revealed that all tested isolates formed biofilms at 24 h, with marked differences among groups (Fig. 1A). The mean biofilm biomass (OD_620_ nm ± SD) was 0.9661 ± 0.0666 for *A. baumannii*, 1.117 ± 0.0611 for *C. albicans*, and 1.433 ± 0.0517 for the co-culture condition, while the negative control wells (TSB only) showed negligible absorbance (0.0709). A one-way ANOVA confirmed a significant difference among the groups (*p* < 0.0001, R^2^ = 0.9852). Post-hoc Šídák’s multiple comparison test revealed that biofilm biomass in the co-culture condition was significantly higher compared to *A. baumannii* alone (mean difference = 0.47, *p* = 0.0001) and *C. albicans* alone (mean difference = 0.32, *p* = 0.0013).

After 48 h, the mean biofilm formation (OD_620_ nm ± SD) was 1.130 ± 0.080 for *A. baumannii*, 1.225 ± 0.075 for *C. albicans*, and 1.640 ± 0.074 for the co-culture (Fig. 1B). A one-way ANOVA revealed a statistically significant difference among the groups (R^2^ = 0.9816, *p* < 0.0001). Post hoc Šídák’s multiple comparisons test confirmed that co-culture resulted in significantly higher biofilm biomass than *A. baumannii* alone (*p* < 0.001) and *C. albicans* alone (*p* < 0.01).

After 72 h, biofilm formation continued to increase across all groups compared to earlier time points (Fig. 1C). The mean biofilm biomass (OD_620_ nm ± SD) was 1.245 ± 0.092 for *A. baumannii*, 1.318 ± 0.069 for *C. albicans*, and higher in the co-culture condition at 1.743 ± 0.075. One-way ANOVA indicated a significant difference among the groups (R^2^ = 0.9825, *p* < 0.0001). Šídák’s multiple comparisons confirmed that co-culture produced significantly greater biofilm biomass than *A. baumannii* alone (*p* < 0.001) and *C. albicans* alone (*p* < 0.01). Together, these results show that the synergistic effect of co-culture on biofilm formation was sustained and further pronounced at 72 h.

#### Time-dependent changes in biofilm biomass

**Fig. 2:**
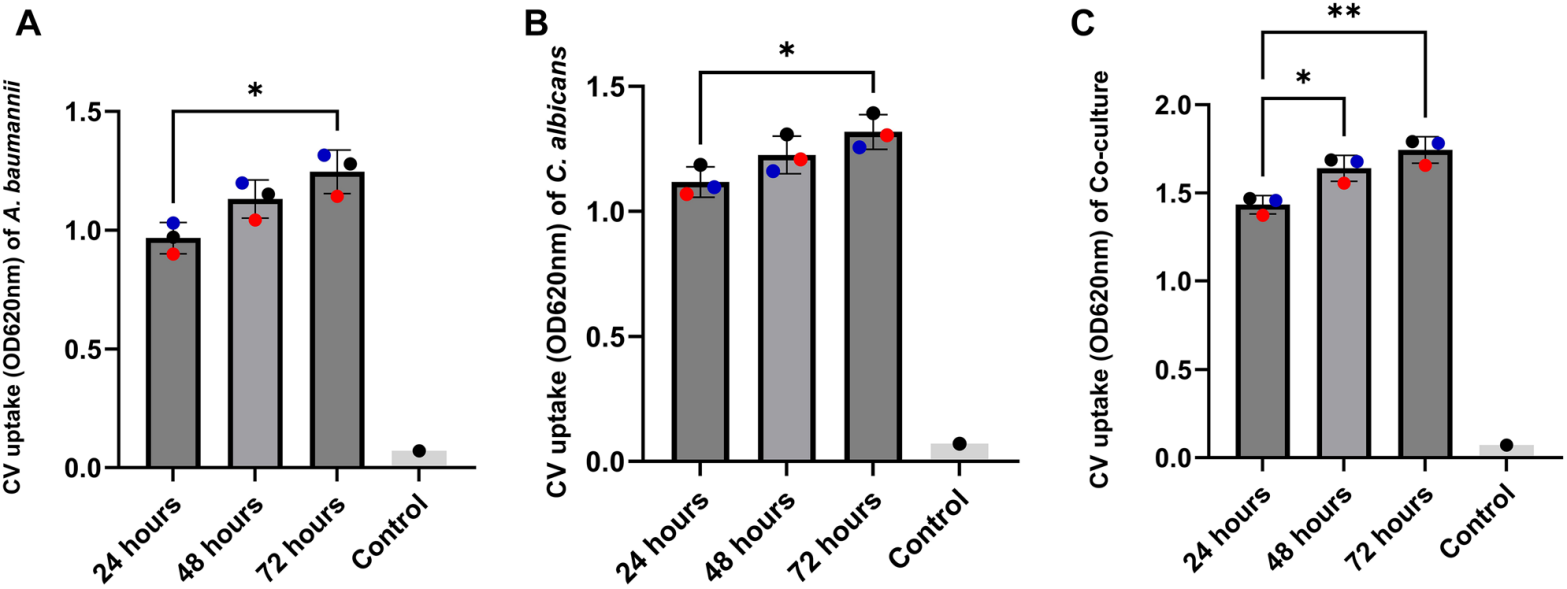
Biofilm biomass of *A. baumannii* and *C. albicans* in mono-culture and co-culture conditions. (**A**) *A. baumannii* biofilm biomass at 24 h, 48 h, and 72 h. (**B**) *C. albicans* biofilm biomass at 24 h, 48 h, and 72 h. (**C**) Co-culture biofilm biomass at 24 h, 48 h, and 72 h. Data represent the mean ± SD of three independent isolates (blue, red, black dots) per group. Statistical analysis was performed using one-way ANOVA followed by Dunnett’s multiple comparison test. Statistical significance is indicated as (*p-value < 0.05, **p-value < 0.01).

This section presents the dynamics of biofilm progression within each condition across different time points. *A. baumannii* showed an increase in biofilm biomass across 24, 48, and 72 h of incubation (Fig. 2A). One-way ANOVA demonstrated a highly significant effect of incubation time on biofilm formation (*p* < 0.0001, R^2^ = 0.966). Dunnett’s multiple comparisons test further revealed that biofilm formation at 72 h was significantly higher than at 24 h (mean difference = – 0.279, *p* = 0.013), while the increase from 24 to 48 h did not reach statistical significance (mean difference = – 0.164, *p* = 0.108).

Moreover, CV biofilm quantification revealed that *C. albicans* exhibited progressive biofilm development over time (Fig. 2B). One-way ANOVA demonstrated a significant overall effect of incubation time on biofilm biomass (*p* < 0.0001, R^2^ = 0.9778). Dunnett’s post hoc analysis showed that biofilm formation at 72 h was significantly higher than at 24 h (mean difference = − 0.2007, *p* = 0.0292). The increase observed between 24 and 48 h was not statistically significant (mean difference = − 0.1083, *p* = 0.2313).

The co-culture model of *A. baumannii* and *C. albicans* showed a progressive increase in biofilm biomass over time (Fig. 2C). One-way ANOVA revealed a highly significant effect of incubation time on biofilm formation (*p* < 0.0001, R² = 0.9880). Post hoc Dunnett’s test showed that biofilm biomass at 48 h was significantly higher than at 24 h (mean difference = − 0.2072, *p* = 0.0239), and a further significant increase was observed at 72 h compared to 24 h (mean difference = − 0.3105, *p* = 0.0035). All experimental groups exhibited significantly greater biofilm biomass than the control (mean difference = 1.362, *p* < 0.0001). These findings indicate that co-culture of *A. baumannii* and *C. albicans* promotes biofilm formation, with a significant increase detectable as early as 48 h and further enhanced at 72 h. On the other hand, mono-cultures showed a gradual pattern.

#### Classification of biofilm-forming ability

Biofilm-forming ability was classified using the cut-off optical density (ODc = 0.0786) derived from negative controls. All tested isolates of *A. baumannii*, *C. albicans*, and their co-culture showed OD isolate > 4xODc (0.0786) at all time points, indicating high biofilm formation. The control group remained within the non-biofilm former (NBF) range. All tested clinical isolates were categorised as high biofilm formers (HBF). Detailed classification data are provided in Supplementary Table S1.

### Gene expression analysis of virulence-associated genes in *A. baumannii* and *C. albicans* during mono-culture and co-culture using qPCR

#### Differential gene expression of (*bap, ompA, and abaI*) genes in *A. baumannii* during mono-culture and co-culture with *C. albicans* at 24 and 48 h

**Fig. 3:**
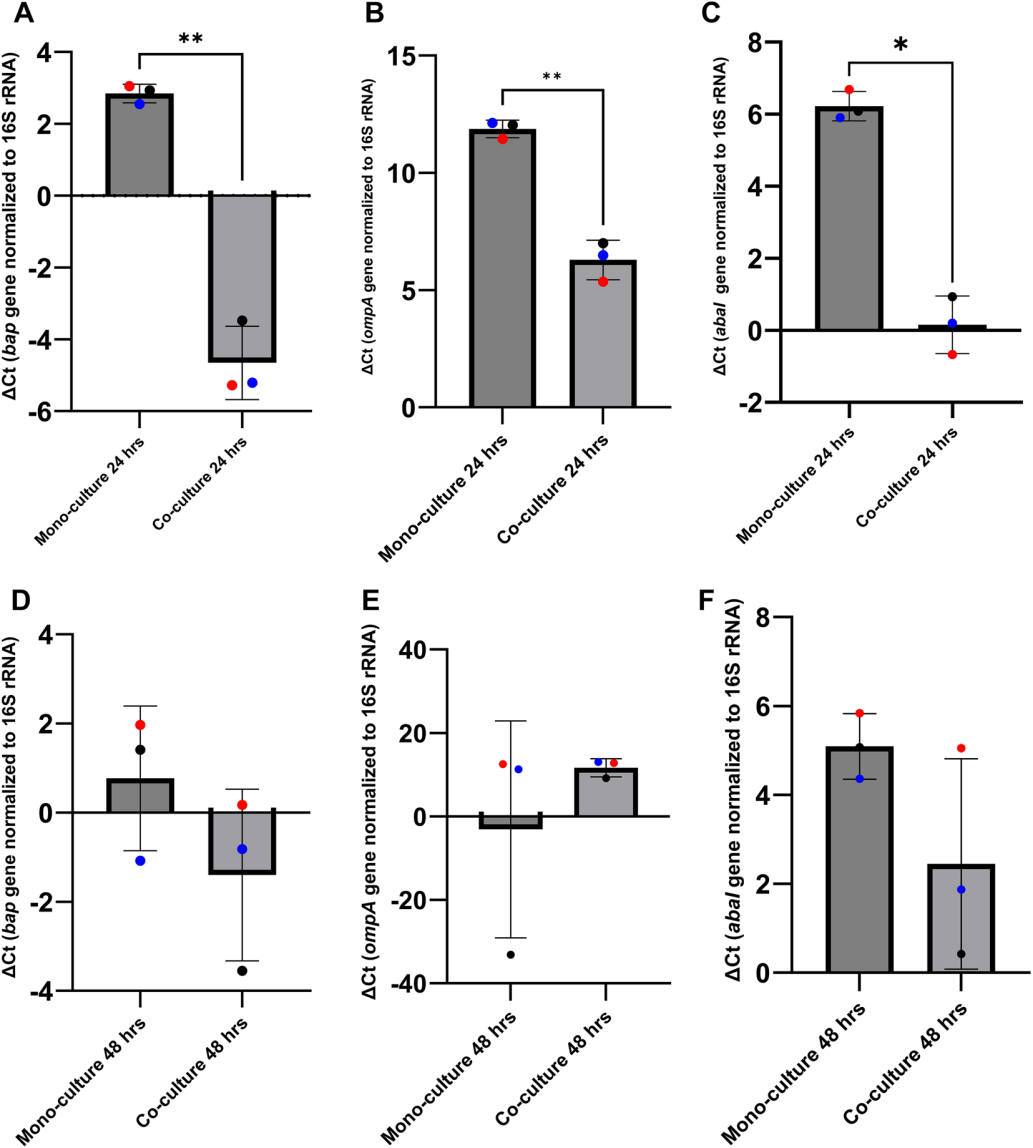
Differential gene expression of (*bap, ompA, and abaI*) genes in *A. baumannii* during mono-culture and co-culture with *C. albicans* at 24 and 48 h. Relative expression of virulence-associated genes (*bap*, *ompA*, and *abaI*) in *A. baumannii* at 24 h and 48 h under mono-culture and co-culture conditions. (**A**–**C**) Gene expression at 24 h. (**D**–**F**) Gene expression at 48 h. Expression levels were analysed by RT-qPCR and normalised to the *16S* rRNA housekeeping gene. Data are presented as ΔCt values, where lower values indicate higher gene expression and vice versa. Each data point represents a single independent isolate, and bars indicate the mean ± SD. Statistical analysis was performed using paired t-tests (*p-value < 0.05, **p-value < 0.01).

qPCR analysis showed a significant difference in *bap* expression between *A. baumannii* mono-culture and co-culture at 24 h. As ΔCt increases, expression decreases. Mono-culture showed low expression (ΔCt mean: 2.84), while co-culture showed reduced ΔCt values (ΔCt mean: – 4.66), indicating strong upregulation. A paired t-test verified significance (mean ± SD difference: – 7.50 ± 0.99, *p* < 0.01) (Fig. 3A). Similarly, *ompA* expression differed significantly at 24 h, with mono-culture showing a ΔCt mean of 11.87, and co-culture 6.28, indicating upregulation. This decrease in ΔCt was statistically significant (mean ± SD difference: – 5.587 ± 0.5220, *p* < 0.01) (Fig. 3B). The quorum-sensing gene *abaI* also showed a significant change: mono-culture ΔCt mean 6.22, co-culture 0.1533, indicating strong upregulation (mean ± SD difference: – 6.067 ± 1.145, *p* < 0.05) (Fig. 3C).

At 48 h, *bap* expression showed no significant difference (mono-culture ΔCt: 0.7666, co-culture: – 1.4; paired t-test: *p* = 0.2896) (Fig. 3D). For *ompA*, showed no significant change (fold changes 0.84 and 0.29. Mean ΔCt values were – 3.1 (mono-culture) and 12.78 (co-culture), with no significant difference (*p* = 0.3955) (Fig. 3E). *abaI* expression also showed non-significant change at 48 h (mono-culture ΔCt: – 5.09, co-culture ΔCt: 2.4466; *p* = 0.1416) (Fig. 3F). The fold changes of *bap, ompA*, and *abaI* were calculated through the 2^–ΔΔCt method^34^. The fold changes are listed in Table 4.

**Table 4:** Fold change in expression of *A. baumannii* virulence genes (Co-culture compared to Mono-culture, 24 and 48 h).

Incubation time	Gene	Isolate 1 (FC)	Isolate 2 (FC)	Isolate 3 (FC)	Mean ± SD
24 h	*bap*	321.80	216.77	85.04	207.87 ± 119.37
	*ompA*	67.65	49.87	32.90	50.14 ± 17.43
	*abaI*	163.14	51.98	35.51	83.54 ± 67.62
48 h	*bap*	3.48	0.84	31.12	11.81 ± 16.36
	*ompA*	0.84	0.29	1.87 × 10⁻^13^	0.38 ± 0.41
	*abaI*	1.73	5.62	25.11	12.43

*Relative fold change in expression *of A. baumannii* virulence genes (*bap, ompA, abaI*) in co- culture compared to mono-culture at 24 and 48 h. Data are presented as fold changes relative to individual patient values and their mean ± SD.

#### Differential gene expression of (*ERG11, ALS3, and HWP1*) genes in *C. albicans* during mono-culture and co-culture with *A. baumannii* at 24 and 48 h

**Fig. 4:**
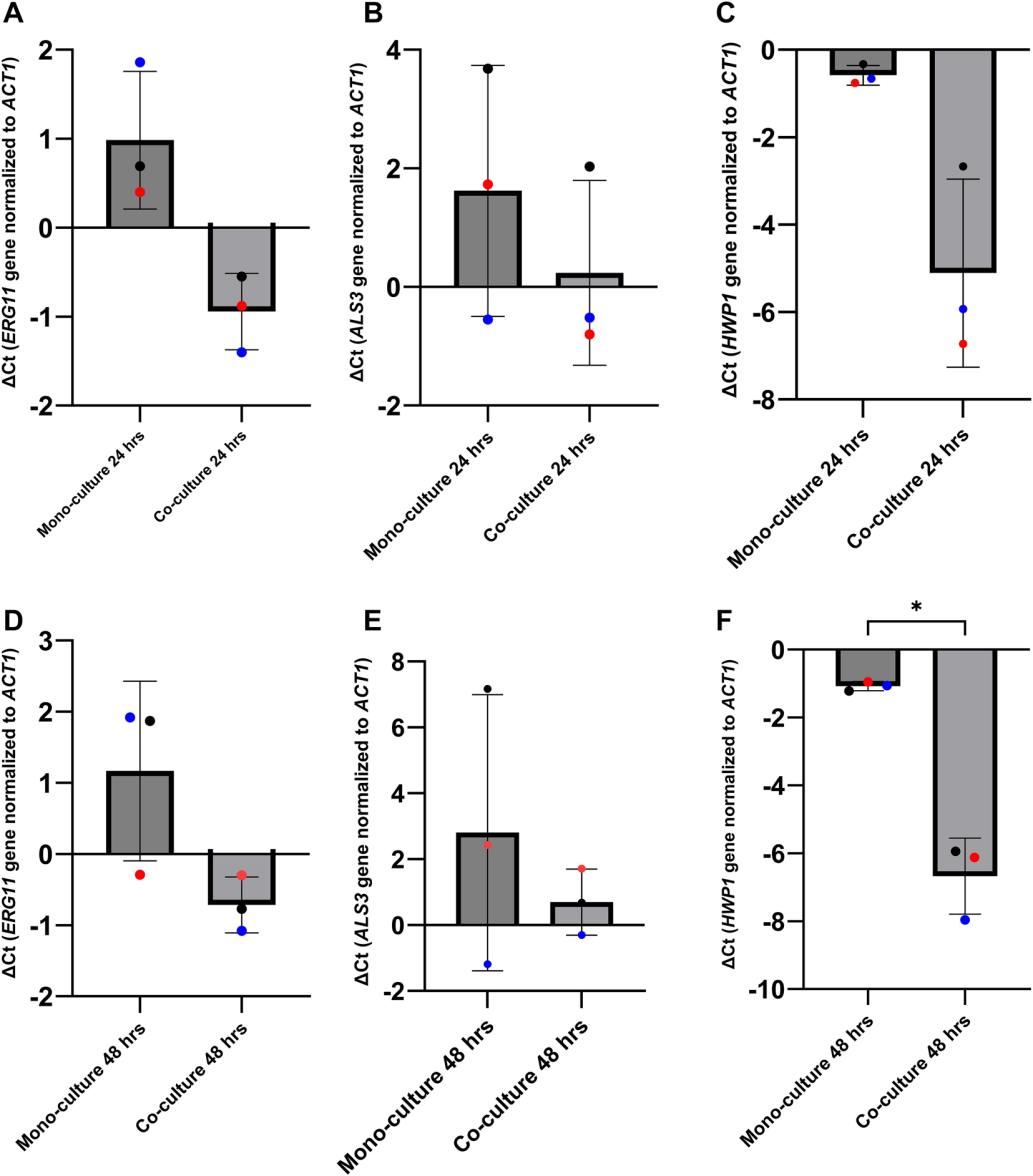
Differential gene expression of virulence genes in *C. albicans* during mono-culture and co-culture with *A. baumannii* at 24 and 48 h. Relative expression of virulence-associated genes (*ERG11*, *ALS3*, and *HWP1*) in *C. albicans* under mono-culture and co-culture with *A. baumannii*. (**A**–**C**) Gene expression at 24 h. (**D**–**F**) Gene expression at 48 h. Expression levels were measured using RT-qPCR and normalised to the *ACT1* housekeeping gene. Data are presented as ΔCt values, where lower ΔCt indicates higher gene expression and vice versa. Each data point represents an individual isolate. Bars represent the mean ± SD. Statistical analysis was performed using paired t-tests (*p-value < 0.05).

At 24 h, *ERG11* in mono-culture showed a ΔCt mean of 0.98, while co-culture showed reduced ΔCt values (ΔCt mean: – 0.94). Paired t-test confirmed that the difference was not statistically significant (mean ± SD difference: – 1.927 ± 1.155, *p* = 0.1018) (Fig. 4A). Similarly, *ALS3* showed a mean ΔCt of 1.62 in mono-culture and 0.24 in co-culture. This decrease in ΔCt was not statistically significant (mean ± SD difference: – 1.383 ± 1.301, *p* = 0.2068) (Fig. 4B). The *HWP1* gene also showed reduced ΔCt values, from –0.58 in the mono-culture to – 5.11 in the co-culture; however, the paired t-test verified that this difference was not statistically significant (mean ± SD difference: – 4.527 ± 1.926, *p* = 0.0554) (Fig. 4C).

At 48 h, *ERG11* expression showed no significant difference (mono-culture ΔCt: 1.17, co-culture: – 0.72; paired t-test: *p* = 0.1837) (Fig. 4D). For *ALS3*, ΔCt values decreased from 2.80 to 0.70, but this change was not statistically significant (mean ± SD difference: – 2.107 ± 3.880, *p* = 0.4463) (Fig. 4E). In contrast, *HWP1* expression showed a statistically significant change at 48 h, with ΔCt decreasing from –1.06 in the mono-culture to – 6.67 in the co-culture, indicating strong upregulation (mean ± SD difference: – 5.597 ± 1.151, *p* < 0.05) (Fig. 4F). The fold changes for *ERG11, ALS3*, and *HWP1* were calculated using the 2^–ΔΔCt method^34^. The fold change values are listed in Table 5.

**Table 5:** Relative gene expression (fold change) of *C. albicans* virulence genes in co-culture versus mono-culture at 24- and 48-h.

Incubation time	Gene	Isolate 1 (FC)	Isolate 2 (FC)	Isolate 3 (FC)	Mean ± SD
24 h	*ERG11*	2.43	9.58	2.36	4.79 ± 4.06
	*ALS3*	5.78	0.98	3.14	3.30 ± 2.41
	*HWP1*	62.68	38.59	5.06	35.44 ± 28.93
48 h	*ERG11*	1.01	8.00	6.23	5.08 ± 3.61
	*ALS3*	1.65	0.54	89.88	30.69 ± 50.47
	*HWP1*	36.00	119.43	26.35	60.59 ± 50.29

*Relative fold change in expression *of C. albicans* virulence genes in co-culture compared to mono-culture at 24 and 48 h. Data are presented as fold changes relative to individual patient values and their mean ± SD.

## Discussion

The existence of polymicrobial communities is supported by their presence in biofilms. Biofilms shield the microbial community through an extracellular matrix that protects it from host immune defense mechanisms, harsh environmental conditions, and antimicrobials. Biofilms could consist of a single species, multiple species, or even different kingdoms. Microbial infections are commonly caused by polymicrobial biofilm, which involves different fungi, bacteria, or viruses^36^. Thus, this study analysed the molecular interactions between *C. albicans* and *A. baumannii,* focusing on virulence and biofilm-related factors in the clinical isolates tested. To our knowledge, most of the previous co-culture studies on different species used laboratory strains or isolates from different patients^37–40^. However, this study explored interactions between co-isolated *C. albicans* and *A. baumannii* to provide an exploratory clinical and physiological perspective despite the limited sample size.

Antimicrobial susceptibility testing was performed using the VITEK-2 system for the co-isolated *C. albicans* and *A. baumannii*. *C. albicans* isolates were susceptible to all antifungal agents tested, including azoles, echinocandins, polyenes, and flucytosine. These results are consistent with previous studies indicating that *C. albicans* isolates generally remain susceptible to commonly used antifungal agents, including azoles and polyenes, particularly in UTI-associated isolates^10,41,42^*.* On the other hand, *A. baumannii* isolates demonstrated extensive drug resistance (XDR). It showed resistance to all tested classes, including β-lactams, carbapenems, aminoglycosides, and fluoroquinolones. This resistance profile is consistent with the studies that emphasized the alarming multidrug resistance capacity of *A. baumannii*^17,43^ Although isolates of *C. albicans* showed susceptibility, their synergistic interaction with *A. baumannii* in biofilms could enhance bacterial and fungal persistence^44^.

Biofilm formation of *A. baumannii* and *C. albicans* individually and in co-culture was assessed by CV assay at 24 h, 48 h, and 72 h. All isolates of *A. baumannii*, *C. albicans*, and their co-culture were high biofilm formers (HBF), with OD values exceeding 4 × ODc at all time points. In co-culture, biofilm biomass increased significantly at all time points compared with each species individually, consistent with a potential synergistic interaction that enhances biofilm development in these isolates. The hyphae of *C. albicans* may serve as scaffolds for bacterial growth, as shown in *Staphylococcus aureus*^45^. They may facilitate *A. baumannii* adherence by presenting *ompA-*binding sites on their hyphal structures, thereby promoting biofilm formation^46^. A significant difference in biofilm biomass between 24 and 72 h was observed for both species, supporting their strong biofilm-forming ability, as in previous studies^47,48^, while no significant difference was observed between 24 and 48 h, indicating the need for extended incubation for biofilm stabilisation^49^. In contrast, co-culture showed a more rapid and pronounced increase in biofilm, with significant differences at 48 and 72 h compared to 24 h, further supporting a synergistic interaction between *A. baumannii* and *C. albicans* in the tested clinical isolates. This high biofilm-forming capacity may contribute to their clinical importance, persistence on catheters, and reduced antimicrobial susceptibility in chronic UTIs. Consequently, patients with polymicrobial UTIs may potentially experience higher biofilm biomass and more complications, which requires further investigation to prove^50^.

The co-culture of *A. baumannii* with *C. albicans* at 24 h showed a statistically significant upregulation of all three biofilm-associated genes compared to mono-culture in the tested clinical isolates. *bap* showed a 207-fold mean upregulation, *ompA* nearly 50-fold, and *abaI* over 80-fold, consistent with a potential synergistic interaction between both species. No previous studies reported such upregulation, while one study showed suppression of *abaI* and *ompA* in *A. baumannii* ATCC 19,606 in the presence of *C. albicans*, suggesting antagonism^51^. Our findings differ from this, as the increased biofilm biomass aligns with upregulation of *bap* and *abaI*, supporting a synergistic interaction. At 48 h, *bap, ompA, and abaI* did not reach statistical significance and showed variation among isolates, reflecting intrinsic heterogeneity in clinical samples.

In *C. albicans*, co-culture with *A. baumannii* at 24 h *showed that ERG11, ALS3, and HWP1* did not reach statistical significance and varied among isolates*,* reflecting intrinsic heterogeneity in clinical samples. This variability was similarly reported in a study showing differential *ALS3* expression among 55 *C. albicans* isolates from septicemia patients^52^. At 48 h, *ERG11* and *ALS3* did not reach statistical significance. However, *HWP1* exhibited a ~ 60-fold increase and statistically significant upregulation relative to monoculture across the tested clinical isolates. This is consistent with the potential that *A. baumannii* enhances hypha-associated virulence.

During co-culture, *A. baumannii* gene expression decreased from 24 to 48 h, possibly due to feedback regulation or nutrient limitation. *C. albicans* may trigger an early burst of *A. baumannii* virulence expression to establish adhesion, followed by downregulation as biofilm maturation progresses. Farnesol secreted by *C. albicans* may modulate *A. baumannii* physiology, though the bacterium’s farnesol efflux pump may confer resistance^40^. In *C. albicans*, co-culture led to reduced *ALS3* and *ERG11* but sustained *HWP1* expression, consistent with a transition from adhesion to hyphal dominance. Persistent *HWP1* upregulation reflects its key role in maintaining hyphal morphology and persistence in polymicrobial environments. This transcriptional pattern corresponds to the observed increase in biofilm biomass in co-culture compared to mono-culture. Collectively, these findings indicate that *C. albicans* exhibits a more invasive phenotype during co-culture with *A. baumannii*, supporting a synergistic interaction that enhances persistence and treatment resistance in UTIs.

To our knowledge, this study provides an exploratory evidence of a synergistic relationship between co-isolates of *A. baumannii* and *C. albicans*, in contrast to previous reports of antagonism^40,53^. However, other studies have reported evidence of synergism between the two pathogens, consistent with this study’s findings. For instance, a study showed that *C. albicans* colonisation is a risk factor for *A. baumannii* ventilator-associated *pneumonia* (VAP); in rat models, fungal colonisation increased *A. baumannii* load and lung damage^51^. Another study suggested that ethanol produced by *C. albicans* enhances *A. baumannii* survival by upregulating efflux pump, phospholipase C, and iron assimilation genes^54^. Furthermore, a previous study suggested that the structural similarity between *A. baumannii ompA* and *C. albicans Hyr1p* suggests potential receptor-like roles in mixed-species biofilm formation^46^. In this exploratory study, it is suggested that there could be a sequential scaffolding mechanism organised by a shift in gene expression, with bacterial virulence genes (*bap, ompA, abaI*) peaking at 24 h, followed by an increase in fungal *HWP1* gene expression at 48 h. The early significant upregulation of *A. baumannii* virulence genes indicates that *A. baumannii* acts as the primary coloniser. While the delay in *HWP1* upregulation at 48 h suggests that *C. albicans* could be responding to the bacterial presence or the quorum-sensing molecules produced by *A. baumannii* at 24 h. The hyphal network produced by *C. albicans* serves as a barrier that protects XDR *A. baumannii*, potentially creating a highly resistant biofilm that is difficult to eradicate in UTI cases.

This study has several limitations. First, the small number of paired clinical isolates may limit the generalizability of the findings. Second, well-characterised reference strains were not included for comparison with the clinical co-isolates. Third, the study evaluated transcriptional changes without assessing corresponding protein expression or functional phenotypes. In addition, gene expression analysis was limited to 24 h and 48 h and did not include the 72 h time point. Finally, antimicrobial susceptibility testing was performed on individual isolates rather than in co-culture.

## Conclusion

In conclusion, this study demonstrates that co-culture of *A. baumannii* and *C. albicans* increases biofilm biomass relative to monocultures, indicating a synergistic interspecies interaction in polymicrobial urinary tract infection models. This effect was accompanied by a time-dependent modulation of virulence gene expression, where *A. baumannii* biofilm–related genes (*bap, abaI,* and *ompA*) were upregulated early at 24 h, whereas the *C. albicans HWP1* gene showed significant upregulation at 48 h. Collectively, these findings suggest a time-dependent modulation of virulence gene expression during co-culture, supporting a potential synergistic interaction between the two pathogens. This exploratory study provides preliminary evidence of bacterial–fungal cooperation that may contribute to enhanced persistence in UTIs. Further studies are required to validate these mechanisms and their clinical implications in polymicrobial infections and treatment resistance.

## Supplementary Information

Below is the link to the electronic supplementary material.

Supplementary Material 1

Supplementary Material 2

Supplementary Material 3

Supplementary Material 4

Supplementary Material 5

Supplementary Material 6

Supplementary Material 7

Supplementary Material 8

Supplementary Material 9

Supplementary Material 10

Supplementary Material 11

Supplementary Material 12

Supplementary Material 13

Supplementary Material 14

Supplementary Material 15

## Data Availability

The datasets used and analyzed during the current study, including raw RT-qPCR Ct values and crystal violet absorbance data, are available from the corresponding author on reasonable request and in supplementary files.

## Abbreviations

*ompA*: Outer membrane protein A
*bap*: Biofilm-associated protein
*abaI*: Acinetobacter autoinducer synthase (quorum-sensing regulator)
*ALS3*: Agglutinin-like sequence 3
*HWP1*: Hyphal wall protein 1
*ERG11*: Lanosterol 14-α-demethylase (ergosterol biosynthesis enzyme, azole resistance marker)
