# Supplementary figures and images for: Exploratory analysis of biofilm formation and virulence gene expression in Acinetobacter baumannii–Candida albicans co-cultured isolates from urinary tract infections
Source: Sci Rep. 2026 Jul 10;16:21635. doi: 10.1038/s41598-026-59824-w (PMC13354557; doi:10.1038/s41598-026-59824-w)

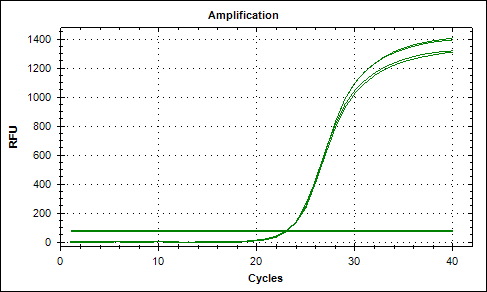

Supplement: Supplementary file 1 — Supplementary Material 1 [file 41598_2026_59824_MOESM1_ESM.png]

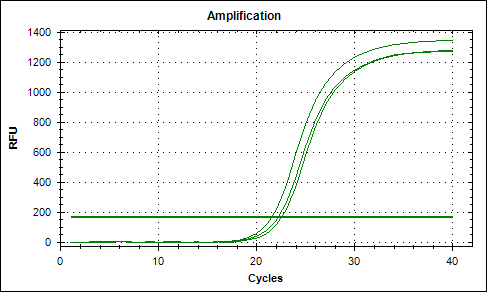

Supplement: Supplementary file 2 — Supplementary Material 2 [file 41598_2026_59824_MOESM2_ESM.png]

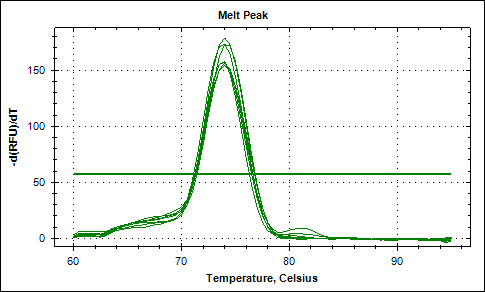

Supplement: Supplementary file 3 — Supplementary Material 3 [file 41598_2026_59824_MOESM3_ESM.png]

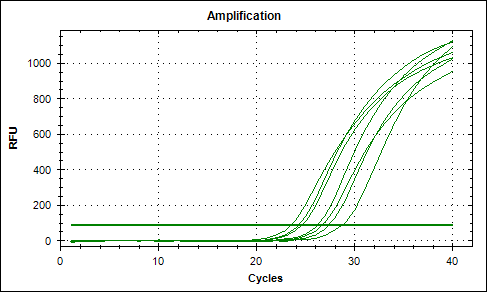

Supplement: Supplementary file 4 — Supplementary Material 4 [file 41598_2026_59824_MOESM4_ESM.png]

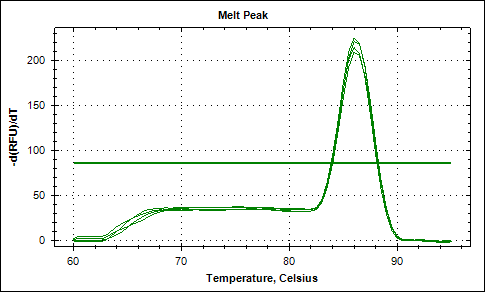

Supplement: Supplementary file 5 — Supplementary Material 5 [file 41598_2026_59824_MOESM5_ESM.png]

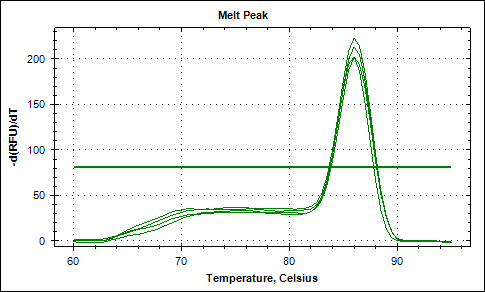

Supplement: Supplementary file 6 — Supplementary Material 6 [file 41598_2026_59824_MOESM6_ESM.png]

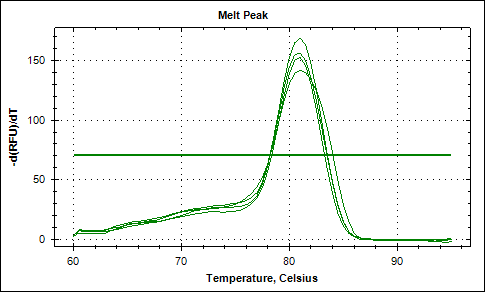

Supplement: Supplementary file 7 — Supplementary Material 7 [file 41598_2026_59824_MOESM7_ESM.png]

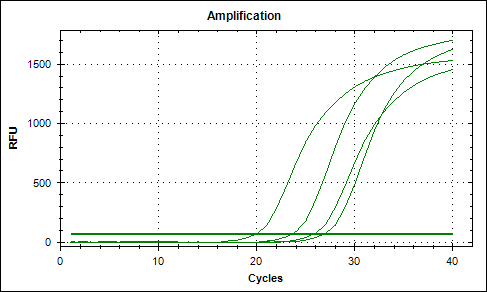

Supplement: Supplementary file 8 — Supplementary Material 8 [file 41598_2026_59824_MOESM8_ESM.png]

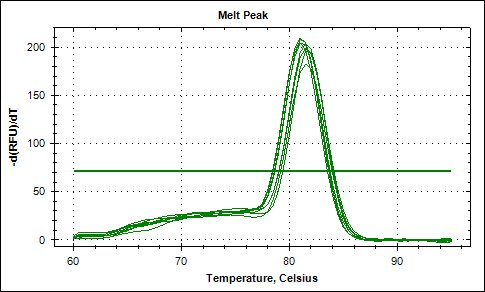

Supplement: Supplementary file 9 — Supplementary Material 9 [file 41598_2026_59824_MOESM9_ESM.png]

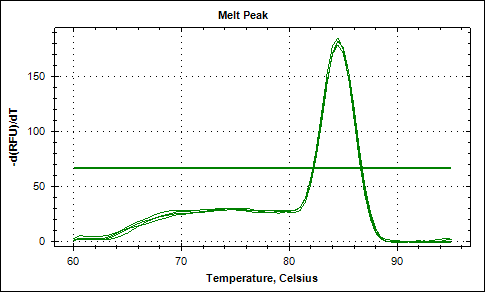

Supplement: Supplementary file 10 — Supplementary Material 10 [file 41598_2026_59824_MOESM10_ESM.png]

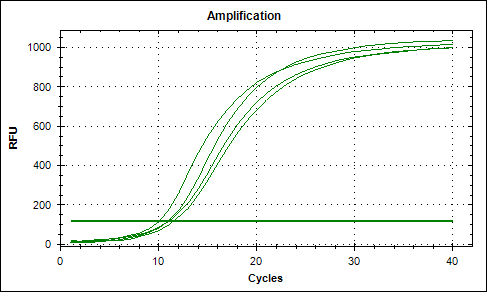

Supplement: Supplementary file 11 — Supplementary Material 11 [file 41598_2026_59824_MOESM11_ESM.png]

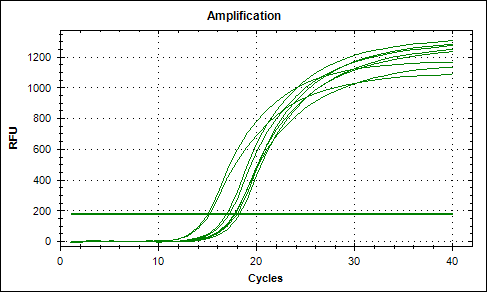

Supplement: Supplementary file 12 — Supplementary Material 12 [file 41598_2026_59824_MOESM12_ESM.png]

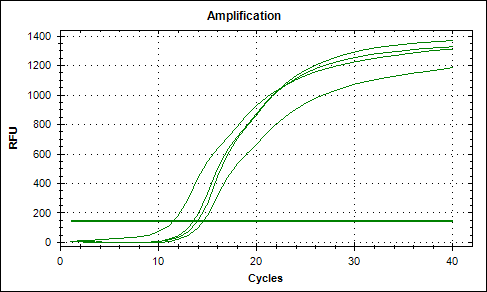

Supplement: Supplementary file 13 — Supplementary Material 13 [file 41598_2026_59824_MOESM13_ESM.png]

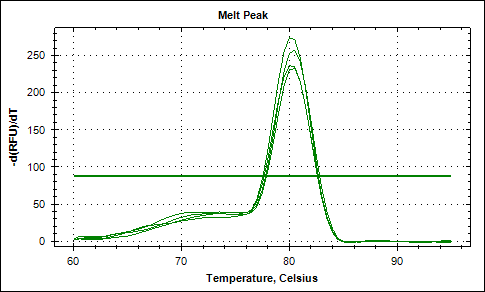

Supplement: Supplementary file 14 — Supplementary Material 14 [file 41598_2026_59824_MOESM14_ESM.png]
