# Supplementary material for: Exploratory analysis of biofilm formation and virulence gene expression in Acinetobacter baumannii–Candida albicans co-cultured isolates from urinary tract infections
Source: Sci Rep. 2026 Jul 10;16:21635. doi: 10.1038/s41598-026-59824-w (PMC13354557; doi:10.1038/s41598-026-59824-w)
Supplement: Supplementary file 15 — Supplementary Material 15 [file 41598_2026_59824_MOESM15_ESM.docx]

**Table S1: Classification of biofilm-forming ability.**

| **Isolates/Condition** | **Time (h)** | **CV values (Mean OD ± SD)** | **CV category** |
| --- | --- | --- | --- |
| *A. baumannii* | 24 | 0.9661±0.0666 | High biofilm former (HBF) |
|  | 48 | 1.130 ±0.0803 |  |
|  | 72 | 1.245 ± 0.091 |  |
| *C. albicans* | 24 | 1.117 ±0.0611 |  |
|  | 48 | 1.225 ±0.0750 |  |
|  | 72 | 1.318 ± 0.0694 |  |
| Co-culture (*A. baumannii + C. albicans)* | 24 | 1.433 ±0.0517 |  |
|  | 48 | 1.640 ± 0.0739 |  |
|  | 72 | 1.743 ± 0.0750 |  |
| Control | - | 0.0709 ± 0.0026 | Non-biofilm former (NBF) |

*ODc = cut-off optical density; NBF = non-biofilm former; HBF = high biofilm former (strong biofilm former). Data are represented as mean ± SD.
